# TRIPLE Score: GPVI and CD36 Expression Predict a Prothrombotic Platelet Function Phenotype

**DOI:** 10.1161/CIRCRESAHA.124.325701

**Published:** 2025-01-22

**Authors:** Alexander P. Bye, Neline Kriek, Carly Kempster, Joanne L. Dunster, Joanne L. Mitchell, Tanya Sage, Suzannah Rawlings, Maria V. Diaz Alonso, Valentina Shpakova, Abigail Whyte, Leanne Dymott, Sharon Mark, Mark Brunton, Joana Batista, Harriet McKinney, Patrick Thomas, Kate Downes, Amanda J. Unsworth, Neil Ruparelia, Charlie McKenna, Chris I. Jones, Jonathan M. Gibbins

**Affiliations:** 1 School of Pharmacy, School of Chemistry Food and Pharmacy (A.P.B.), University of Reading, United Kingdom; 2 School of Biological Sciences and Institute for Cardiovascular and Metabolic Research, (A.P.B., N.K., C.K., J.L.D., T.S., S.R., M.V.D.A., V.S., N.R., C.I.J., J.M.G.), University of Reading, United Kingdom; 3 Institute of Cardiovascular Sciences, College of Medical and Dental Sciences, University of Birmingham, United Kingdom (J.L.M.).; 4 University Department of Cardiology, Royal Berkshire Hospital, Reading, United Kingdom (A.W., L.D., S.M., M.B., N.R., C.M.).; 5 Department of Haematology, University of Cambridge, Cambridge Biomedical Campus, United Kingdom (J.B., H.M.K., P.T., K.D.).; 6 National Health Service Blood and Transplant, Cambridge Biomedical Campus, Cambridge, United Kingdom (K.D.).; 7 Discovery and Translational Science Department, Leeds Institute of Cardiovascular and Metabolic Medicine, University of Leeds, United Kingdom (A.J.U.).; 8 Cardiology Department, Hammersmith Hospital, Imperial College London, United Kingdom (N.R.).

**Keywords:** blood platelets, collagen, phenotype, platelet activation, platelet function tests, risk, thrombosis

Thrombosis remains a leading cause of death globally.^1^ Platelet reactivity and responsiveness to antiplatelet drugs are highly variable, impairing accurate dosing of antithrombotic therapies and resulting in increased bleeding and residual risk of cardiovascular events. The link between high platelet reactivity and cardiovascular events is well established,^2^ and recent studies investigating guided antiplatelet therapy have reported encouraging outcomes, particularly in the context of percutaneous coronary interventions.^3^ However, logistics and infrastructure limit routine assessment of platelet reactivity using specialist aggregometry methods. We present the TRIPLE (Thrombotic Reactivity Indicator using Platelet GPVI, CD36 [cluster of differentiation 36] Expression, and age) Score, which predicts platelet hyperreactivity associated with increased in vitro thrombus formation on the basis of easily measured biomarkers, eliminating the need to measure platelet function. The TRIPLE Score presents a novel method for stratifying patients based on a platelet function phenotype that could enable the identification of high-risk patient populations for personalization of antiplatelet therapy. We first identified platelet function parameters associated with increased thrombus formation using an in vitro model of arterial thrombosis (Figure [A]) in a healthy cohort (Figure [Ai]). A 6-fold difference in the propensity to generate large thrombi on a standardized thrombotic stimulus (type I collagen) was observed across the cohort (Figure [Aii and Aiii]). Comprehensive platelet function analysis was performed using the PPAnalysis platform^4^ and thrombus formation was found to correlate significantly with platelet sensitivity (analogous to EC_50_) to the GPVI (glycoprotein VI) agonist CRP-XL (crosslinked collagen-related peptide) (Figure [Aiv]), indicating that CRP-XL sensitivity may provide a useful biomarker of thrombus formation.

**Figure.**
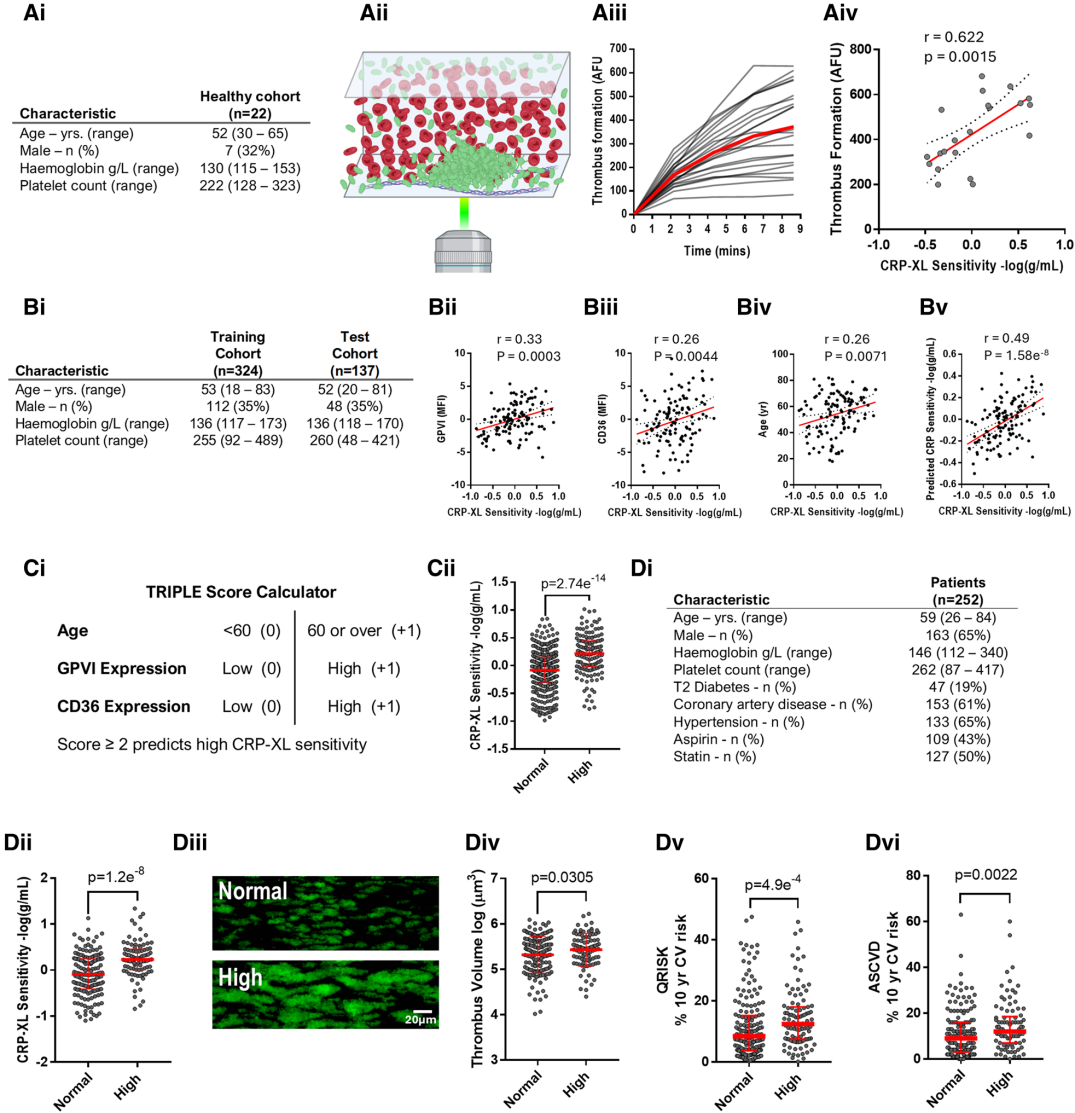
**GPVI (glycoprotein VI) and CD36 expression predict a prothrombotic platelet function phenotype. Ai**, Healthy participant characteristics (n=22). **Aii**, Schematic created in Biorender.com of fluorescence imaging of DiOC6 (3,3′-Dihexyloxacarbocyanine iodide) labeled platelet thrombus growth on collagen during perfusion of whole blood at 1000 s^−1^. **Aiii**, Quantification of real time in vitro thrombus formation in arbitrary fluorescence units (AFU) (n=22, gray lines) and the mean response (red line). **Aiv**, Linear regression of end-point thrombus formation against CRP-XL (crosslinked collagen-related peptide) sensitivity (derived from the EC_50_ of CRP-XL-evoked platelet CD62P [P-Selectin] exposure assessed by flow cytometry). **Bi**, Characteristics of training and test cohorts. Linear regression of CRP-XL sensitivity against (**Bii**) GPVI expression, (**Biii**) CD36 (cluster of differentiation 36) expression, (**Biv**) age, and (**Bv**) predicted CRP-XL sensitivity for the test cohort. **Ci**, The TRIPLE (Thrombotic Reactivity Indicator using Platelet GPVI, CD36 Expression, and Age) Score predicts high CRP-XL sensitivity for scores of 2 or above, where age (≥60 years), GPVI expression (upper 50%), and CD36 expression (upper 25%) each score 1. **Cii**, CRP-XL sensitivity for normal and high TRIPLE Scores for the whole cohort. **Di**, Patient characteristics under investigation for coronary artery disease recruited at the Royal Berkshire Hospital. **Dii**, CRP-XL sensitivity, (**Diii**) representative thrombus images, (**Div**) end-point thrombus volume, (**Dv**) QRISK, and (**Dvi**) ASCVD (atherosclerotic cardiovascular disease) scores (age, 40–75 years only) for normal and high TRIPLE score patients. Bars are median±interquartile range. Normality assessed using Shapiro-Wilk test and analysis performed using the Student *t* test (**Cii** and **Dii**) or Mann-Whitney *U* test (**Div** through **Dvi**). Linear regression plots display *r* and *P* values from Pearson (**Bii**, **Biii**, and **Bv**) or Spearman (**Biv**) correlations. Statistical analyses performed using R or GraphPad Prism, version 7.05.

To develop a platelet function phenotype stratification strategy, we investigated biomarkers associated with CRP-XL sensitivity in 461 healthy volunteers (Figure [Bi]). Bidirectional stepwise linear regression was used to determine the optimal combination from a panel of platelet surface proteins with established roles in platelet function (integrin α_2_, α_2b_ [CD41a], α_2b_ [CD41b], β1, β3, CD9 [cluster of differentiation 9], CD36, CD148 [cluster of differentiation 148], CD151 [cluster of differentiation 151], GPIb [glycoprotein Ib], GPV [glycoprotein V], GPVI [glycoprotein VI], GPXI [glycoprotein XI], and PECAM-1 [platelet endothelial cell adhesion molecule-1]) and volunteer characteristics (age, sex, platelet count, and mean platelet volume) to predict CRP-XL sensitivity in a randomly selected training cohort (n=319). A 3-parameter model utilizing age plus GPVI and CD36 platelet surface expression gave optimal explanatory power (*R*^2^=0.24 and mean absolute error [MAE]=0.27). The GPVI receptor mediates platelet activation responses to collagen, while CD36 is a scavenger receptor that evokes activation in response to multiple stimuli. Validation of these parameters in the test cohort (n=137), demonstrated that these parameters correlated independently (GPVI versus CD36, *P*=0.940; GPVI versus age, *P*=0.270; CD36 versus age, *P*=0.459; Spearman correlation) with sensitivity for CRP-XL (Figure [Bii through Biv]) and a multiple linear regression model utilizing the 3 parameters more accurately predicted CRP-XL sensitivity (Figure [Bv]).

To standardize stratification using these biomarkers, we developed the TRIPLE Score in which age (≥60 years) GPVI, expression (above population median), and CD36 expression (upper quartile) each score 1, and a total score ≥2 predicts high CRP-XL sensitivity (Figure [Ci]). TRIPLE Scores were calculated for the total healthy cohort. Participants predicted to have high CRP-XL sensitivity had significantly greater measured CRP-XL sensitivity compared with participants predicted to have normal sensitivity (Figure [Cii]).

To validate the TRIPLE Score for stratifying patients based on platelet reactivity, blood samples were collected from patients (n=252, exclusions: patients<18 years, with prior acute coronary syndrome (ACS) within 12 months or prescribed anticoagulant medications) undergoing investigation for suspected coronary artery disease (Figure [Di]). The TRIPLE Score predicted that 36% of patients would have high CRP-XL sensitivity. These patients were found to have significantly greater measured sensitivity to CRP-XL compared with patients predicted to have normal sensitivity (Figure [Dii]). Notably, patients predicted to have high CRP-XL sensitivity also formed significantly larger thrombi in vitro (Figure [Diii and Div]). Cardiovascular risk scores utilized in the United Kingdom (QRISK3) and the United States (ASCVD) were significantly higher for patients with high TRIPLE Scores (Figure [Dv and Dvi]).

In summary, we have developed and validated the TRIPLE Score to predict high sensitivity to the GPVI agonist CRP-XL, which is associated with a prothrombotic platelet function phenotype. Evidence linking platelet reactivity and cardiovascular events continues to emerge^5^ and our findings indicate that GPVI hyperreactivity is specifically linked to thrombus formation and that the development of novel antiplatelet agents targeting the CD36 and GPVI receptors could offer a new therapeutic strategy for patients not effectively managed by current antiplatelet therapies. The TRIPLE Score utilizes protein expression to identify high reactivity, bypassing the technical demands of platelet function testing which will enable platelet function prediction to be incorporated into CVD (cardiovascular disease) risk scores and clinical practice.

## ARTICLE INFORMATION

### Acknowledgments

Schematics were created with BioRender.com.

### Sources of Funding

This study was supported by grants from the British Heart Foundation (PG/16/36/31967, RG/15/2/31224 and RG/20/7/34866) and a Rosetrees Trust Translation Research Fellowship Award.

### Disclosures

The authors report no conflicts. Data associated with this study are available from the corresponding author upon reasonable request.

Blood collection was approved by the University of Reading Research Ethics Committee (UREC20/20) and Cambridge East Research Ethics Committee (Genes and Platelets REC 10/H0304/65). Patients were recruited at the Royal Berkshire Hospital’s University Department of Cardiology (Project ID285583, Rec Reference 20/NW/0364). All subjects provided informed consent in accordance with the Declaration of Helsinki.
